# Expression Profiling of Ribosome Biogenesis Factors Reveals Nucleolin as a Novel Potential Marker to Predict Outcome in AML Patients

**DOI:** 10.1371/journal.pone.0170160

**Published:** 2017-01-19

**Authors:** Virginie Marcel, Frédéric Catez, Caroline M. Berger, Emeline Perrial, Adriana Plesa, Xavier Thomas, Eve Mattei, Sandrine Hayette, Pierre Saintigny, Philippe Bouvet, Jean-Jacques Diaz, Charles Dumontet

**Affiliations:** 1 Cancer Research Center of Lyon, UMR INSERM 1052 CNRS 5286, Centre Léon Bérard, Lyon, France; 2 Université Lyon 1, Lyon, France; 3 Nuclear domains and pathologies team, Cancer Cell Plasticity Department, Lyon, France; 4 Anticancer antibodies team, Immunity, Microenvironment and Virus Department, Lyon, France; 5 Department of Biology, Hospices Civils de Lyon, Centre Hospitalier Lyon Sud, Pierre Bénite, France; 6 Department of Hematology, Hospices Civils de Lyon, Centre Hospitalier Lyon Sud, Pierre Bénite, France; 7 Department of Medecine, Centre Léon Bérard, Lyon, France; 8 Ecole Normale Supérieure de Lyon, Lyon, France; Wayne State University, UNITED STATES

## Abstract

Acute myeloid leukemia (AML) is a heterogeneous disease. Prognosis is mainly influenced by patient age at diagnosis and cytogenetic alterations, two of the main factors currently used in AML patient risk stratification. However, additional criteria are required to improve the current risk classification and better adapt patient care. In neoplastic cells, ribosome biogenesis is increased to sustain the high proliferation rate and ribosome composition is altered to modulate specific gene expression driving tumorigenesis. Here, we investigated the usage of ribosome biogenesis factors as clinical markers in adult patients with AML. We showed that nucleoli, the nucleus compartments where ribosome production takes place, are modified in AML by analyzing a panel of AML and healthy donor cells using immunofluorescence staining. Using four AML series, including the TCGA dataset, altogether representing a total of about 270 samples, we showed that not all factors involved in ribosome biogenesis have clinical values although ribosome biogenesis is increased in AML. Interestingly, we identified the regulator of ribosome production nucleolin (*NCL*) as over-expressed in AML blasts. Moreover, we found in two series that high *NCL* mRNA expression level was associated with a poor overall survival, particular in elderly patients. Multivariate analyses taking into account age and cytogenetic risk indicated that *NCL* expression in blast cells is an independent marker of reduced survival. Our study identifies *NCL* as a potential novel prognostic factor in AML. Altogether, our results suggest that the ribosome biogenesis pathway may be of interest as clinical markers in AML.

## Introduction

Acute myeloid leukemia (AML), which results from hematopoietic stem cell disorders, is a heterogeneous disease. Indeed, AML is associated with a high diversity of molecular alterations promoting blast transformation [1,2]. Consequently, multiple factors influence AML patients’ outcome that lead to a high heterogeneity of clinical outcomes in AML disease. Treatment choice is currently based on patient stratification into different risk groups that helps to distinguish between favorable, intermediate and poor risk groups [2]. Classification systems have evolved from a purely morphological stratification (French-American-British (FAB)) to more recent systems, which incorporate cytogenetic data and age of patient at diagnosis (World Health Organization (WHO) or European LeukemiaNet (ELN)) [2–5]. In spite of these stratification criteria, only 35–40% of AML patients under 60 years and less than 10% of elderly AML patients (over 60 years) can be cured [2,6]. The lack of power of the current classifications indicates that cytogenetics and age at diagnosis are not sufficient to accurately predict AML outcome. To improve patient management and help identify novel therapeutic strategies in patient subgroups, a major issue in AML patient care is to identify novel biomarkers, including aberrant gene expression [2,7,8].

In neoplastic cells, the high proliferation rate of tumor cells is sustained by an increased ribosome biogenesis due to hyper-activation of RNA polymerase I (RNA *pol* I) associated with an increase in protein synthesis [9–11]. However, only few studies have investigated the usage of ribosome biogenesis factors as clinical markers in cancer. In AML, blasts have been shown to contain a higher number of nucleoli, the site of ribosome biogenesis, than control cells using AgNOR staining (silver-staining of Nucleolar Organizer Region-related proteins), suggesting an increase in ribosome biogenesis [12,13]. The only ribosome biogenesis factor currently known to be altered in AML is nucleophosmin 1 (NPM1), which is mutated in 40% of AML patients and is associated with favorable prognosis [1,2,14]. However, the effects of these mutations on NPM1 nucleolar functions have never been investigated. Interestingly, recent studies have reported the direct contribution of ribosome biogenesis in hematopoietic stem cell biology, suggesting that changes in expression of ribosome biogenesis factors could affect blast proliferation and differentiation and thus be used as original clinical markers in AML [15–17].

Here, we investigated for the first time the clinical significance of a panel of factors involved in ribosome biogenesis. We focused our study on genes encoding factors regulating either ribosome production, such as nucleolin (*NCL*) and fibrillarin (*FBL*), or ribosome composition—such as *FBL*, *NOP56*, *NOP58* and *NHP2L1*. NCL and FBL are two of the most abundant nucleolar proteins playing a central role in rDNA transcription by regulating activity of RNA *pol* I [18–20]. In addition, FBL together with *NOP56*, *NOP58* and *NHP2L1* constitute the rRNA 2’-O-ribose methylation complex whose pattern alterations have been shown to modulate gene expression [21–23].

## Materials and Methods

### Patient samples

Bone marrow smears were collected from healthy donors (controls, n = 4) and AML patients (n = 6) at initial diagnosis to analyze number of nucleoli per cell and nucleoli morphology by immunofluorescence. In addition, RNA samples of five series (controls, series 1, series 2, series 3 and TCGA series) issued from bone marrow or blood samples were used to investigate clinical value of ribosome biogenesis factors (see characteristics in Table 1). Smears, controls, series 1, series 2 and series 3 were collected at Hôpital Lyon-Sud (HCL, Lyon, France). AML samples corresponded to patients with *de novo* AML from initial diagnosis with a minimum of 15% of blast cell count. Control samples were issued from healthy donors selected as potential allograft donors. Series 3 was constituted of AML samples from series 1 and 2 for which clinical data were available (follow-up of survival patients up to 5 years). The TCGA series was extracted from a public database hosting datasets issued from the Cancer Genome Atlas project (TCGA Research Network, Acute Myeloid Leukemia dataset). All samples were obtained at time of diagnosis with written informed consent at corresponding hospitals with approval of local ethics committees (Comité d’Ethique de Lyon) that approved this study. Characterization of the classical cytogenetic markers was determined by the corresponding hospitals. Clinical annotations were available for the series 3 and TCGA (S1 Dataset).

**Table 1:** Patient characteristics.

			Controls	Series 1	Series 2	Series 3	Series TCGA
Total samples (n)			9	45	126	59	113
Period of samples collection			2011–2013	2002–2007	2011–2013	2002–2013	2001–2010
**Age**	**All**	**number**	4	40	122	59	113
		**minimum age**	25	23	2	23	18
		**maximum age**	73	108	93	94	88
		**Mean (SE)**	46.7 (10.4)	59.7 (2.5)	58.0 (1.9)	61.5 (2.3)	55.5 (1.5)
	**< 60years**	**number (%)**	3 (75%)	22 (55%)	53 (43.4%)	26 (44.1%)	59 (52.2%)
		**minimum age**	24	23	2	23	18
		**maximum age**	51	59	59	58	59
		**Mean (SE)**	37.8 (7.7)	48.9 (2.0)	37.9 (2.1)	44.2 (1.9)	43.3 (1.5)
	**≥60 years**	**number (%)**	1 (25%)	18 (45%)	69 (56.6%)	33 (55.9%)	54 (47.8%)
		**minimum age**	73	61	60	63	60
		**maximum age**	73	108	93	94	88
		**Mean (SE)**	73.0 (0.0)	72.9 (2.6)	73.5 (9.0)	75.2 (1.2)	68.9 (0.9)
**Sex**	**Male**	**n (%)**	0	17 (37.8%)	63 (50%)	33 (55.9%)	63 (57.8%)
	**Female**	**n (%)**	0	21 (46.7%)	60 (47.6%)	26 (44.1%)	50 (44.2%)
	**Unknown**	**n (%)**	9 (100%)	7 (15.5%)	3 (2.4%)	0	0
**Samples origin**	**Bone marrow**	**n (%)**	9 (100%)	7 (15.6%)	98 (77.8%)	42 (71.2%)	113 (100%)
	**Blood**	**n (%)**	0	37 (82.2%)	24 (19.0%)	9 (15.3%)	0
	**Unknown**	**n (%)**	0	1 (2.2%)	4 (3.2%)	8 (13.6%)	0

### Immunofluorescence and quantification

Air dried bone marrow smears were hydrated with 1X PBS, fixed with 4% formaldehyde and permeabilized with 0.5% Triton X-100. Immunostaining was performed using a rabbit anti-FBL antibody at 1/500 dilution (ab5821, Abcam), a mouse anti-NCL antibody at 1/2000 ([4E2] ab 13541, Abcam) and AlexaFluor 488^***®***^/AlexaFluor 555^***®***^ conjugated secondary antibody (Molecular Probes). Following immunostaining, nuclei were stained with Hoechst 33342 (Molecular Probes), and smears were mounted with Fluoromount G mounting medium (Electron Microscopy Sciences). Images were collected with a x63 NA 1.4 Plan-Apochromat objective on a Carl Zeiss LSM 780 confocal microscope. Image analysis and quantification were performed on wide-field images collected with a x40 NA 1.30 Plan Fluor objective on a Nikon NiE microscope equipped with an Orca Flash 4 camera (Hamamatsu), and using the NIS Elements software (Nikon). Briefly, nuclei were segmented using intensity thresholding of Hoechst signal, and saved as individual ROI (Region Of Interest). Nucleoli were segmented using intensity thresholding of FBL signal within the ROIs and their number and size were extracted.

### High throughput qPCR

Total RNAs were extracted by Trizol Reagent and retro-transcribed using M-MLV RT enzyme (Invitrogen). Gene expression was quantified by real-time qPCR using Sybr Green chemistry and specific primer sets (Table A in S1 File). Classical qPCR was performed with Light Cycler 480 II using 2X SYBR Green Master Mix (Roche). High throughput qPCR was performed with BioMark HD System (Fluidigm) using Master Mix 2X EvaGreen (Bio-Rad) following a step of pre-amplification as described by the manufacturer (Fluidigm). Fold-changes were calculated using the 2^-ΔΔCT^ method using *GAPDH* gene expression levels as normalizer (S1 Fig) and one control sample as a normalizer among the different plates [4,5,24,25]. Each sample was retro-transcribed twice independently and each RT products were quantified in triplicate at least in two independent runs of qPCR. Some genes were not amplified in some samples of series 1 and series 2 due to technical problems and the available number of samples varies from gene to gene.

### Analysis of TCGA AML dataset

The Cancer Genome Atlas (TCGA) was queried (last query in July 2014) (The Cancer Genome Atlas—Data Portal. Available at https://tcga-data.nci.nih.gov/tcga/tcgaHome2.jsp). Clinical and pathological information from only 113 patients with AML were downloaded. Indeed, some AML cases lack information about patients’ survival and AML cases with history of neoadjuvant treatment were excluded from the analysis since chemotherapeutic drugs have been shown to inhibit ribosome biogenesis that may thus affect expression of factors involved in ribosome biogenesis [26]. Level 3 RNAseqv2 data were downloaded, corresponding to gene read counts that were generated from the IlluminaHiSeq platform and normalized using MapSplice to do the alignment and RSEM to perform the quantification [27,28]. Then a log2 transformation was performed.

### Statistical analyses

Statistical analyses were performed using GraphPad Prism 5.0a (GraphPad Software, Inc). Median comparison between two unpaired groups was performed using the non-parametric Mann-Whitney test. Correlations between different methods or primers sets were tested using the non-parametric Spearman r test. Overall survival was measured from the date of diagnosis to death or censored at the last follow-up. Prognostic value of the different genes was estimated using the Kaplan-Meier method and the Log-Rank Mantel-Cox model was used to assess statistical significance. A cut-off based on data dispersion was used instead of an absolute cut-off since quantification of mRNA levels was measured using two different methods (RT-qPCR and RNA-seq). In particular, the 75^th^ percentile was used as a cut-off for all the datasets to defined “low” versus “high” expression since we observed that *NCL* is over-expressed in AML patients and that *NCL* median levels in AML patients correspond to the highest *NCL* levels in healthy donors. This strategy allowed the usage of a standardized, common method to determine the cut-offs among the different data sets issued from distinct methods. A multivariate Cox regression survival analysis was performed taking into account *NCL* expression, age at diagnosis and cytogenetic stratification to determine whether *NCL* is an independent marker of prognosis. All *P*-values corresponded to two-tailed *P*-values. *P*-values <0.05 were considered statistically significant.

## Results

### Nucleoli number and architecture are modified in AML samples

**Fig 1:**
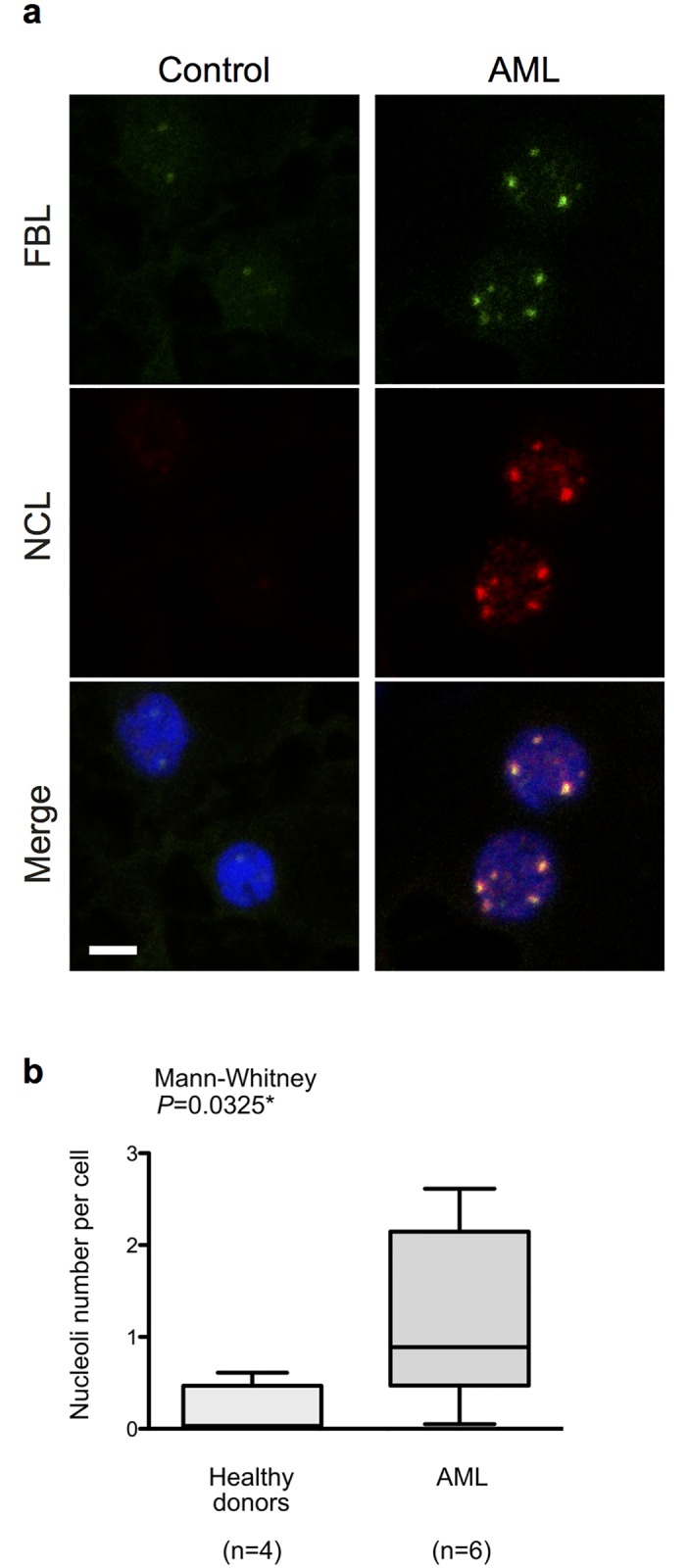
Increase in nucleoli number and size in AML patients. (**A**) Immunofluorescence staining of nucleolar fibrillarin (FBL) and nucleolin (NCL) in healthy donors (control) and AML patients bone marrow smears. FBL (green) and NCL (red) patterns are shown individually in top and middle images, and merge image with nuclei staining (blue) is shown in the bottom. Images were collected using confocal microscope (scale = 10μm). (**B**) Number of nucleoli per cell, quantified by image analysis from 4 control and 6 AML smears. Box and whisker plots represents median (middle horizontal bar), inter-quartile range (bottom and top of the box) and minimal/maximal values (bottom and top of the whiskers) of nucleoli number per cell. Representative panels of images used to perform these image analyses for each individual are shown in S2 Fig. n: number of samples; *: *P*<0.05.

In a first instance, we investigated whether nucleoli number and organization are modified in AML patients. The morphological aspect of nucleoli reflects ribosome biogenesis activity and correlates with the neoplastic status of cells [13]. To investigate changes in nucleoli number and morphology in AML patient bone marrow cells, we performed immunofluorescence staining of FBL and NCL, two of the most widely used marker of nucleoli, on fresh bone marrow smears from 4 healthy donors and 6 AML patients at initial diagnosis. Several pre-staining and unmasking treatments were assayed and PBS re-hydration coupled with formaldehyde fixation provided an optimal staining of nucleoli. FBL and NCL staining appeared dramatically different in control versus AML patient samples (Fig 1A and S2 Fig). The FBL and NCL nucleolar signal was weak or almost undetectable in healthy donor samples (from 0 to 2 nucleoli per cell, S3A Fig). In normal bone marrow cells, the lack of detectable nucleolar structure is consistent with previous analysis of normal bone marrow analyzed by electron-microscopy or AgNOR staining and is indicative of a low nucleolar activity [29,30]. In contrast, the staining was strong with numerous spots in most AML patient samples (Fig 1A and S2 Fig). Nucleoli appeared more numerous in AML bone marrow as compared with control samples, although the number of nucleoli per cell varied from cell to cell (a mean number from 0 to 3.5 nucleoli per cell, S3 Fig). In addition, the size of nucleoli was also larger, and many cells displayed irregularly shaped nucleoli (for example, see S2 Fig, patient AML1). Changes in nucleolar size were confirmed by measuring the surface of nucleoli in each individual samples exhibiting nucleoli (S3B Fig). Altogether, our data show that nucleoli organization is significantly altered in AML patient bone marrow cells and further support that ribosome biogenesis is increased in AML cells compared to healthy cells.

### NCL and NOP56, two factors involved in ribosome biogenesis, are over-expressed in AML patients

Since nucleoli number and size were increased in AML blasts, we investigated whether some factors of ribosome biogenesis were over-expressed in AML samples. We collected a control series of healthy donors and two AML series from initial diagnosis (series 1 and 2) (Table 1). Gene expression was quantified by high throughput qPCR using microfluidic technology (Fluidigm system devices). This technology allows to analyze expression of 48 genes in 48 samples in a single run and thus to reduce the amount of samples required. In our hand, a strong statistical correlation was observed between data generated by high throughput qPCR and classical qPCR quantification (S4A Fig) as already shown by others [31,32]. Due to the diversity of sample origins, being issued either from bone marrow or blood (Table 1), we confirmed that expression of genes of interest was not tissue-dependent (Table B in S1 File). Finally, in series 1 and 2, *C-Myc* and *NPM1* genes were significantly over-expressed in AML samples compared to controls (Table 2 and S4B and S4C Fig). These results are in agreement with previously published data [33–36], thus supporting the relevance of the series 1 and 2 to study gene expression in AML.

**Table 2:** Mean comparison of gene expression between controls and AML patients.

	Series 1					Series 2				
	Controls		AML patients		Mann-Whitney	Controls		AML patients		Mann-Whitney
	n	Mean^$^ (SE)	n	Mean^$^ (SE)	*P*-value#	n	Mean^$^ (SE)	n	Mean^$^ (SE)	*P*-value#
**C-Myc**	7	2.233 (0.41)	21	4.165 (0.30)	0.0058**	7	2.233 (0.41)	20	6.883 (0.51)	0.0004***
**NPM1**	8	1.418 (0.93)	32	3.213 (0.42)	0.0759	8	1.418 (0.93)	75	9.900 (0.50)	< 0.0001***
**18S**	8	0.341 (0.41)	25	0.888 (0.31)	0.1722	8	0.341 (0.41)	34	2.833 (0.28)	0.0007***
**NCL**	9	1.372 (0.60)	25	2.944 (0.34)	0.0386*	9	1.372 (0.60)	90	7.474 (0.41)	< 0.0001***
**FBL**	8	2.029 (0.65)	31	3.257 (0.27)	0.0536	8	2.029 (0.65)	84	1.883 (0.18)	0.8789
**Nop58**	8	1.535 (0.63)	15	3.860 (0.53)	0.0155*	na	na	na	na	na
**Nop56**	8	2.571 (0.78)	28	4.241 (0.38)	0.0316*	8	2.571 (0.78)	98	10.07 (0.40)	< 0.0001***
**NHP2L1**	5	-1.35 (2.27)	16	1.678 (0.58)	0.0759	5	-1.35 (2.27)	90	1.929 (0.16)	0.0105*

^$^ Mean value: log2(fold-change);

^#^ Two-tailed P-value; ns: no significant; na: not available

*: P-value < 0.05;

**: P-value < 0.01;

***: P-value < 0.001

We first compared expression of some ribosome biogenesis factors between controls and AML patients using a Mann-Whitney test in series 1. In particular, we analyzed the expression of *18S* rRNA together with that of *NCL* and *FBL*, these three genes reflecting ribosome synthesis, as well as the expression of the components of the rRNA 2’-O-ribose methylation complex (*FBL*, *NOP58*, *NOP56* and *NHP2L1*). Analysis of series 1 showed that only *NCL*, *NOP58* and *NOP56* genes are significantly over-expressed in AML samples compared to controls (Table 2).

**Fig 2:**
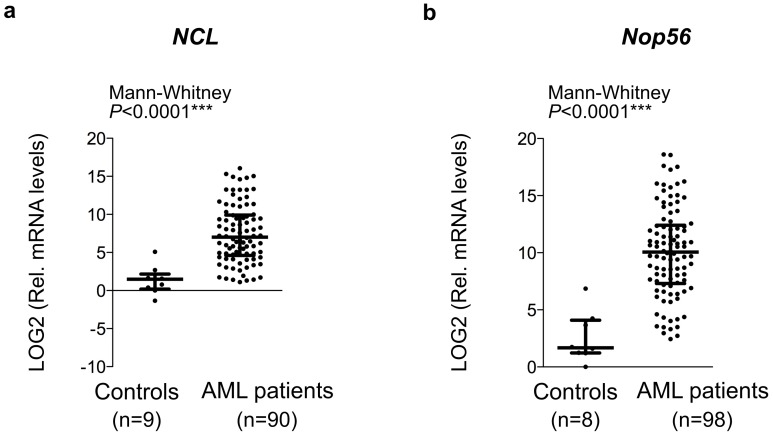
Over-expression of *NCL* and *NOP56*, two ribosome biogenesis factors, in AML blasts. (**A-B**) Mean comparisons of *NCL* (A) and *NOP56* (B) gene expression between controls and AML patients were compared using the Mann-Whitney test. Log_2_ (Relative RNA levels) were determined using high-throughput qPCR in series 2. Graphs represents median (middle horizontal bar) and inter-quartile range (bottom and top bars) calculated on the log_2_ (Relative RNA levels) of each individual sample (grey dot). n: number of samples; ***: *P*<0.0001.

We then validated these results in series 2. Similarly to results obtained in series 1, a significant over-expression of *NCL* and *NOP56* was observed in AML patients as compared to controls (Table 2 and Fig 2). Moreover, a significant higher expression of *18S* and *NHP2L1* was observed in AML samples in series 2, although with lower p-values than *NCL* and *NOP56*. Thus, comparison of analysis of both the series 1 and 2 identified *NCL* and *NOP56* as the genes involved in ribosome biogenesis, which are the most significantly over-expressed in AML blasts.

To rule out that the above conclusion was dependent upon primer design, similar analyses were performed using a second set of primers to quantify *NCL* and *NOP56* gene expression. As expected, the second set of NCL and Nop56 primers showed a significant, positive correlation with the first primer sets (S5A and S5B Fig). The use of a second set of NCL and Nop56 primers to analyze series 1 and 2 confirmed the over-expression of both *NCL* and *NOP56* in AML samples (S5C–S5H Fig).

Altogether, our analyses in the two series using two independent primer sets demonstrated that *NCL* and *NOP56* are over-expressed in AML patients compared to controls.

### High NCL mRNA level is associated with poor prognosis

**Fig 3:**
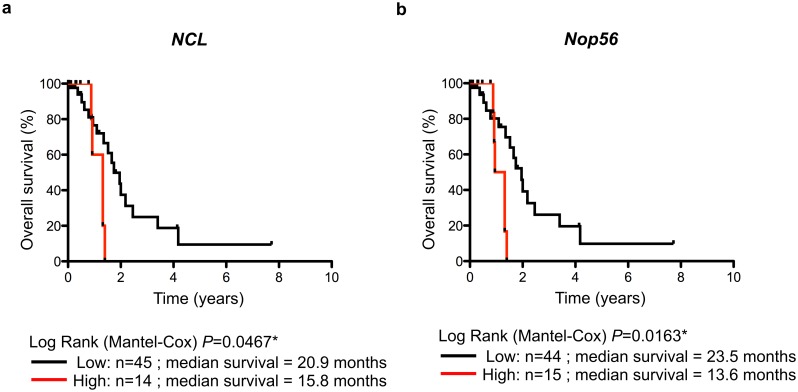
Overall survival of AML patients related to *NCL* and *NOP56*. (**A-B**) Kaplan-Meier analysis of overall survival rates (event = death related to AML disease) according to *NCL* (A) and *NOP56* (B) expression at diagnosis in series 3. The data are dichotomized at the 75% percentile value into high and low mRNA level groups. n: number of samples; *: *P*<0.05.

Due to the over-expression of *NCL* and *NOP56* in AML, we tested the prognostic impact of *NCL* and *NOP56* on patient outcome. To do so, a third AML series was compiled using AML patients from series 1 and 2 for whom clinical annotations were available (series 3, see Table 1 for characteristics). The 75^th^ percentile was used to dichotomize gene expression in two groups, “low” and “high” *NCL* expression levels since (1) the highest levels of *NCL* expression in healthy donors correspond to about the *NCL* median levels in AML patients (Fig 2A), (2) *NCL* is over-expressed in AML patients compared to healthy donors (Table 2), and (3) the fold-changes of *NCL* displayed one of the largest dispersion (S6A Fig). No absolute cut-off was used since mRNA levels were quantified using either RT-qPCR (series 3) or RNA-seq (see below, TCGA series). In series 3, Kaplan-Meier and univariate Cox regression analyses identified two genes whose expression is associated to AML patient outcome (Table C in S1 File). We first observed a significant association between high expression levels of *NCL* and overall survival of AML patients (Fig 3A). Similar significant association was observed using a lower cut-off value corresponding to the median (P = 0.064**) (data not shown). In addition, high *NOP56* expression level was also significantly associated with poor AML outcome (Fig 3B).

**Fig 4:**
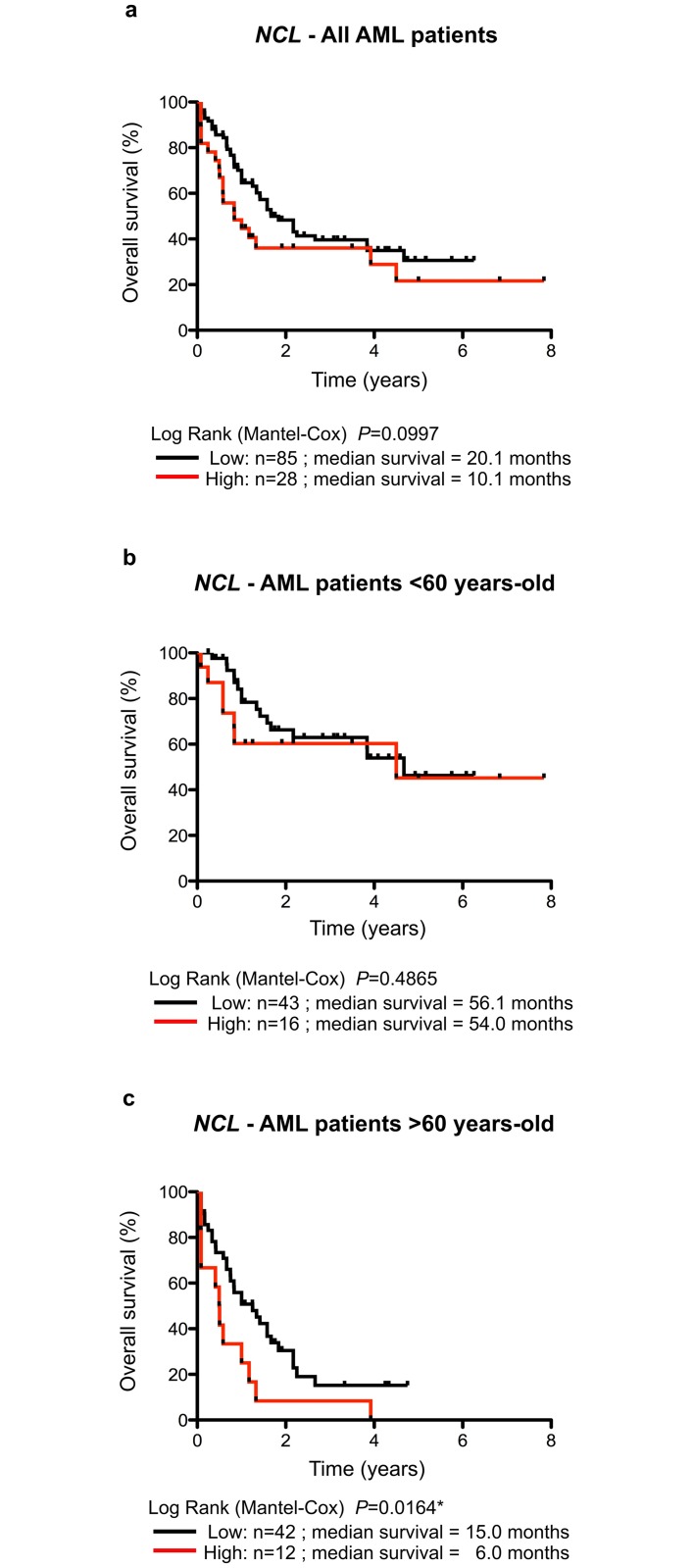
Overall survival of AML patients related to *NCL* expression depending upon age in the TCGA series. (**A-C**) Kaplan-Meier analysis of overall survival rates (event = death related to AML disease) according to *NCL* expression in series TCGA for all AML patients (A), AML patients younger than 60 years (B) or AML patients older than 60 years (C). The data are dichotomized at the 75% percentile value into high and low mRNA level groups. n: number of samples; *: *P*<0.05.

To validate the use of *NCL* and *NOP56* expression in predicting AML outcome, an additional, independent series was studied (series TCGA, see Table 1). We extracted expression profiles of the seven genes of interest (*C-Myc*, *NPM1*, *NCL*, *FBL*, *NHP2L1*, *NOP56* and *NOP58*) determined by RNA-seq of AML samples from the TCGA dataset (TCGA Research Network, Acute Myeloid Leukemia dataset). Filters were used to restrict the analysis to primary AML samples thus allowing the collection of 113 samples to build the validation series. Dichotomization of expression levels was performed using the 75^th^ percentile as a cut-off to conserve similar strategy within the study (S6B Fig). Using the TCGA dataset, no significant association was observed between *NOP56* expression levels and AML patient’s outcome (Table D in S1 File). In contrast, a borderline significant association was observed between high *NCL* expression levels and poor prognosis in series TCGA (Fig 4A and Table D in S1 File). Despite of the borderline significance, these data showed the same tendency than using series 3 –high *NCL* levels being associated with poor AML prognosis. Interestingly, none of the five additional genes of interest (*C-Myc*, *NPM1*, *FBL*, *NHP2L1*, *NOP58*) showed association with AML patient outcome, supporting the specificity of *NCL* as prognostic marker in AML (Table D in S1 File).

The lack of strict concordance in series 3 and TCGA in terms of prognostic impact of *NCL* could result from the significant differences in the mean age of AML patients between the two series (P-value = 0.023*) (Table 1). Indeed, AML patients in series 3 had a median age at diagnosis, which was 6 years greater than for patients in the TCGA series. Therefore, to determine whether *NCL* expression could stratify patient risk depending on their age at diagnosis, we re-analyzed the prognostic impact of *NCL* expression in the TCGA series taking into account the age of patients (Table D in S1 File). Such analyses cannot be addressed using series 3 due to the small number of samples (Table 1). In TCGA samples, a clear difference in prognostic impact of *NCL* expression was observed depending on patient age. In AML patients diagnosed before 60 years old, overall survival is similar in patients expressing either high or low levels of *NCL* in their bone marrow (Fig 4B). In contrast, high *NCL* expression level is significantly associated with poor prognosis in elderly AML patients (more than 60 years old) (Fig 4C). Elderly AML patients expressing high *NCL* expression levels in their bone marrow showed a median survival of 6 months while those expressing low *NCL* expression levels had a median survival of 15 months (Log Rank Mantel-Cox, *P* = 0.0164). *NCL* expression levels could therefore be used as a prognostic factor for survival in elderly AML patients.

In addition to age, cytogenetic alterations is an important risk factor [1,2,6,14]. However, the main issue with the cytogenetic risk classification resides in the stratification of AML patients belonging to the intermediate and poor risk groups since these two groups have almost similar outcomes [4,5,25]. This finding was confirmed in the TCGA dataset (S7A Fig). We thus investigated the use of *NCL* expression in improving cytogenetic risk classification in AML patients of the TCGA series using Kaplan-Meier and Cox regression analyses. While *NCL* expression had no significant impact on patient outcome in the favorable and poor risk groups, enhanced *NCL* expression was associated with shorter overall survival in the intermediate group (S7B Fig and Table A in S1 File). Overall, our data suggest that *NCL* expression could help in identifying prognostic subgroups within the current cytogenetic risk classification.

Finally, we investigated whether NCL is an independent marker of prognosis in AML patients using the TCGA series. We performed a multivariate analysis to adjust possible confounding variables (age at diagnosis, cytogenetic alteration and *NCL* mRNA levels) on all AML samples issued from the TCGA dataset (n = 113). The best model associated with poor overall survival contained three independent markers: age at diagnosis (*P* = 0.00012***); cytogenetic alterations (*P* = 0.0170*); and *NCL* mRNA levels (*P* = 0.0230*). These analyses demonstrated that *NCL* expression is associated with poor overall survival independently of these other common prognostic factors. Overall, our data indicate that *NCL* expression is a novel prognostic marker of survival in AML.

## Discussion

Because of the need for improving the current risk classification in order to improve AML patient management, and given the emerging role of ribosomes in controlling HSC differentiation and HSC-derived diseases [1,2,15,17,37], we investigated the clinical significance of factors involved in ribosome biogenesis in AML to identify novel useful biomarkers [2]. Comparison of immunofluorescence staining of the nucleolar markers FBL and NCL between control and AML fresh bone marrow smears revealed that nucleoli numbers and shapes are altered in AML patients at diagnosis. The observed difference could result either from a higher representation of myeloid progenitors, or from an alteration of nucleolar organization in AML blast compared to normal progenitor cells. Additional studies will be required to evaluate nucleoli modifications specifically in AML blast. We found that a large fraction of cells in normal bone marrow and only a small fraction of AML cells display no detectable FBL and NCL by immunofluorescence. Although a technical issue cannot be fully excluded, the low detection of nucleolar structure in bone marrow cells is consistent with previous analysis of bone marrow smears analyzed by electron-microscopy or AgNOR staining, and is indicative of a very low nucleolar activity in these cells [2–5,29,30]. Overall, our data are compatible with previous studies based on AgNOR staining [2,6,13,38], and consolidate the observation that ribosome biogenesis is altered in AML disease. The increase in nucleoli count in AML cells is indicative of a higher ribosome biogenesis activity, and is consistent with the proliferative status of AML blasts. Using two independent sets of primers and three AML series at diagnosis, we identified *NOP56* and *NCL* as two ribosome biogenesis factors over-expressed in AML patients compared to healthy individuals. It is worth to note that other ribosome biogenesis factors (FBL, NHP2L1, NPM1) are not significantly over-expressed. This observation supports the specificity of over-expression of *NCL* and *NOP56* ribosome biogenesis factors in AML disease and suggests that *NCL* and *NOP56* may have important and additional functions in ribosome biogenesis that are necessary to promote acute myeloid leukemogenesis.

Although we clearly identified *NOP56* as being over-expressed in AML samples, the prognostic significance of *NOP56* remains to be clarified. Indeed, we observed a significant association between high *NOP56* expression levels and poor outcome of AML patients in the test series 3 that was not confirmed in the validation series TCGA. Few studies investigated either the clinical significance or the biological role of *NOP56* in hematological disorders. *NOP56* expression is increased in Burkitt’s lymphoma-associated C-Myc mutants and its expression promotes wild-type C-Myc-induced cell transformation and increases tumor size, suggesting a link between B-cell lymphoma and *NOP56* [2,7,8,39]. Based on the role of *NOP56* in hematopoietic-related disorders, use of larger data sets will be required to clearly conclude concerning the clinical significance of *NOP56* in AML.

In contrast to *NOP56*, our data clearly demonstrated that *NCL* expression has a prognostic value in AML outcome. We first demonstrated that *NCL* is over-expressed in AML patients compared to controls. Shen and collaborators recently showed using analysis of previously published GEO data sets (2 series of 54 AML younger than 15 year-old and 26 AML patients) that *NCL* mRNA expression levels is statistically higher in AML patients than in healthy donors [9–12]. The over-expression of *NCL* in AML is supported by our observation that NCL staining is higher in AML bone marrow smears than in controls (Fig 1A). In addition, a study has shown that comparison of NCL expression by immunohistochemistry in normal and blast cells within the bone marrow of AML patients revealed more abundant NCL staining in blasts than in healthy cells [12,13,40]. Thus, these data support the over-expression of *NCL* in AML. At present, regulation of *NCL* expression remains largely unknown and mechanism that drives *NCL* over-expression in AML patients remains to be deciphered. Using both training series 3 and validation TCGA series, we demonstrated that high *NCL* expression is associated with poor AML outcome. More importantly, our multivariate analyses suggest that *NCL* is an independent marker of poor prognosis when taking into account age at diagnosis and cytogenetic alterations. Preliminary analyses suggest that AML samples with high expression levels of *NCL* mRNAs mainly correspond to acute myeloblastic leukemia with maturation (FAB subtypes M1 and M2) (P = 0.0137**) (data not shown), however such observation cannot explain the association between high expression of *NCL* and poor prognosis of AML patients. Interestingly, none of the five additional genes of interest showed association with AML patient outcome, supporting the specificity of *NCL* as prognostic marker in AML. Using a GEO data set of 86 AML patients, Shen et *al*. also reported a significant association between high *NCL* mRNA levels and poor prognosis in AML patients [1,2,12,14]. However, no information was given regarding age or cytogenetic status of AML patients neither regarding its clinical value as an independent marker of AML prognosis. Our data and the ones of Shen and collaborators strongly support that *NCL* is a novel prognostic factor in AML. The stratification using *NCL* expression levels allows to discriminate two groups in elderly AML patients (> 60 years)–low and high expression-related groups of *NCL* expression associated in TCGA series with median survivals of approximately 15 or 6 months, respectively. However, these results cannot be validated using the series 3 due to the small sample number or using additional data sets that are currently unavailable. Ederly AML patients are more often treated with “curative intent” when the cytogenetic profile is favourable and receive palliative or experimental therapy when the cytogenetic profile is unfavourable [41–43]. Stratification of elderly AML patients using *NCL* could thus be useful to guide therapy choices in these particular patient subgroups. Establishment of prospective series enriched in elderly AML patients with information regarding their current treatment regimen will be required to firmly conclude about the utility of *NCL* to improve current risk classifications.

The best characterized activity of NCL remains its role in regulating ribosomal RNA synthesis [15–18]. Indeed, NCL has FACT activity (Facilitates Chromatin Transcription), modifies the epigenetic status of rDNA genes and thus enhances ribosome synthesis. In addition, NCL has a key role in HSC biology and HSC-derived diseases. It has been shown that NCL regulates expression of CD34 thus participating in the maintenance of transcriptional program defining HSCs [18–20,44]. Moreover, recent studies have reported that knocking down NCL in AML cellular models decreases cell colony formation and reduces tumor growth in xenograft models, supporting a key role of NCL in acute myeloid leukemogenesis [12,21,23]. Interestingly, this latter report showed that knocking-down NCL is associated with a decrease in global DNA methylation that could result from DNMT1 down-regulation induced by NCL in this AML model [12,13]. Recent data suggest that DNA methylation profiles are markers of AML patients’ outcome and segregate current risk classifications [29,30,45,46]. Thus, the identification of NCL as a marker of poor AML outcome could be closely related to the epigenetic activity of NCL. Moreover, usage of AS-1411, an oligodeoxynucleotide aptamer specifically targeting NCL that reached phase I and II clinical trials for cancer treatment including AML, displayed similar effects as NCL knock-down [12,31,32]. Based on the current knowledge of NCL in promoting acute myeloid leukemogenesis and on the ongoing efforts to develop agents specifically targeting NCL, our study support the usage of NCL as a novel prognostic marker of AML outcome with potent implications for NCL as a novel therapeutic target in AML

Several data suggest that ribosome biogenesis contributes to hematopoietic stem cell disorders (HSCs), such as AML [9,15–17,33–37]. In addition, mutations in NPM1 have been identified in AML patients and are associated with favorable prognosis for patients without cytogenetic alterations although the impact of such mutation on nucleolar functions of NPM1 remain to be investigated [1,2,6,14]. In this study, we identified two factors involved in ribosome that are over-expressed in AML patients, *NOP56* and *NCL*. More importantly, expression of one of them, *NCL*, predicts AML outcome, independently of the current risk factors—age at diagnosis and cytogenetic alterations. Altogether, our study supports the usage of this ribosome biogenesis factor as clinical markers. It opens up an original and yet unexplored pathway, the ribosome biogenesis pathway, in clinic. In particular, NCL could represent a novel opportunity to improve management of elderly AML patients, not only as an original and independent prognostic factor useful to guide therapy choices but also as novel therapeutic target to develop innovative strategy dedicated to a particular elderly patients subgroup.

## Supporting Information

S1 Dataset Expression and clinical data of human samples. (XLS)

S1 Fig Comparison of expression of different internal reference genes for relative gene quantification using qPCR. Ct values of three internal reference genes, *GAPDH* (A), *ACTB* (B) and *18S* (C), were compared to identify the most suitable housekeeping gene in our experimental conditions. Three major criteria were required: first, the absence of significant difference between controls and AML patients; second, the smallest coefficient of variation; and third, a mean CT of about 20 that corresponds to the mean CT of most of our genes of interest. Only *GAPDH* gene matched these criteria and was thus used as normalizer.
(TIFF)

S2 Fig Detection of nucleoli in cells of control and AML patient's bone marrow smears. Immunofluorescence staining of nucleolar FBL and NCL on control (top panel) and AML (bottom panel) patient's bone marrow smears. FBL (green) and NCL (red) pattern are shown individually in top and middle images, and merged image with nuclei staining (blue) is shown in the bottom image. Images were collected using confocal microscopy (scale = 10μm).
(TIFF)

S3 Fig Increased number and size of nucleoli in AML patient’s bone marrow smears. (**A**) Quantification and analysis of nucleoli number by image analysis from 4 control and 6 AML bone marrow smears. The number of nucleoli per cell was measured using FBL signal in each samples and ranged from 0 to 4. The median number of nucleoli per cell was represented for each individual samples. No nucleolus was observed in the control Ind4. (**B**) Quantification and analysis of nucleoli size by image analysis from 4 control and 6 AML bone marrow smears. When nucleoli are detected within a sample, the nucleolus size was measured using FBL signal in each samples. The median size of nucleoli per cell was represented for each individual samples. No nucleolus was observed in the control Ind4. Box and whisker plots represent median (middle bar in the box), interquartile range (bottom and top of box) and minimal/maximal values (bottom and top whisker). The number of cells or nucleoli analyzed for each samples are indicated in bracket. Representative panels of images used to perform these image analyses are shown in Fig 1 and S1 Fig.
(TIFF)

S4 Fig *C-Myc* but not *NPM1* is over-expressed in AML patients. (**A**) Validation of high-throughput qPCR method using microfluidic system device. Correlation between fold-changes calculated from Ct values determined by classical RT-qPCR and high-throughput qPCR was investigated on few samples for *C-Myc* and *NPM1* gene using Spearman test. (**B-C**) Expression of *C-Myc* and *NPM1* genes in AML patients. Mean comparison of *C-Myc* (B) and *NPM1* (C) gene expression between controls and AML patients was investigated using Mann-Whitney test in serie 1 analyzed by classical RT-qPCR. Graphs represents median (middle horizontal bar) and interquartile range (bottom and top bars) calculated on the Log2(Relative mRNA levels) of each individual samples (grey dot). n: number of samples; *: *P*<0.05; **: *P*<0.01; ***: *P*<0.001.
(TIFF)

S5 Fig Over-expression of *NCL* and *Nop56* in AML patients. (**A-B**) Correlation between log_2_ (Relative mRNA levels) issued from two different sets of primers to analyze expression of *NCL* (A) and *Nop56* (B). Correlation was determined using Spearman test. (**C-H**) Mean comparison of *NCL* and *Nop56* gene expression between controls and AML patients using Mann-Whitey test. Relative RNA levels of *NCL* (C, E, G) and *Nop56* (D, F, H) were determined using high-throughput qPCR in series 1 (C-F) and series 2 (G-H) with two different sets of primers (set-1: C, D; set-2: E-G, F-H). Graphs represents median (middle horizontal bar) and interquartile range (bottom and top bars) calculated on the log_2_ (Relative mRNA levels) of each individual samples (grey dot). n: number of samples; *: *P*<0.05; ***: *P*<0.0001.
(TIFF)

S6 Fig Expression distribution of markers of ribosome biogenesis in AML patients. (**A**) Expression distribution of the different genes analyzed by high-throughput qPCR in series 3. (**B**) Expression distribution of the different genes analyzed by RNA-seq in series TCGA. Box and whisker plots represent median (middle bar in the box), interquartile range (bottom and top of box) and minimal/maximal values (bottom and top whisker). Grey: gene usually over-expressed in AML samples; green: genes coding proteins regulating rRNA synthesis; red: genes coding proteins of the rRNA methylation complex.
(TIFF)

S7 Fig Overall survival of AML patients related to *NCL* in intermediate group of cytogenetic risk classification in TCGA series. (**A-B**) Kaplan-Meier analysis of overall survival rates (event = death related to AML disease) according to cytogenetic risk classification (A) and to *NCL* in intermediate group of cytogenetic risk classification in AML patients (B). The data are dichotomized at the 75% percentile value into high and low mRNA level groups. n: number of samples.
(TIFF)

S1 File Supporting File including Supporting Tables. (DOCX)
