# Supplementary figures and images for: Expression Profiling of Ribosome Biogenesis Factors Reveals Nucleolin as a Novel Potential Marker to Predict Outcome in AML Patients
Source: PLoS One. 2017 Jan 19;12(1):e0170160. doi: 10.1371/journal.pone.0170160 (PMC5245884; doi:10.1371/journal.pone.0170160)

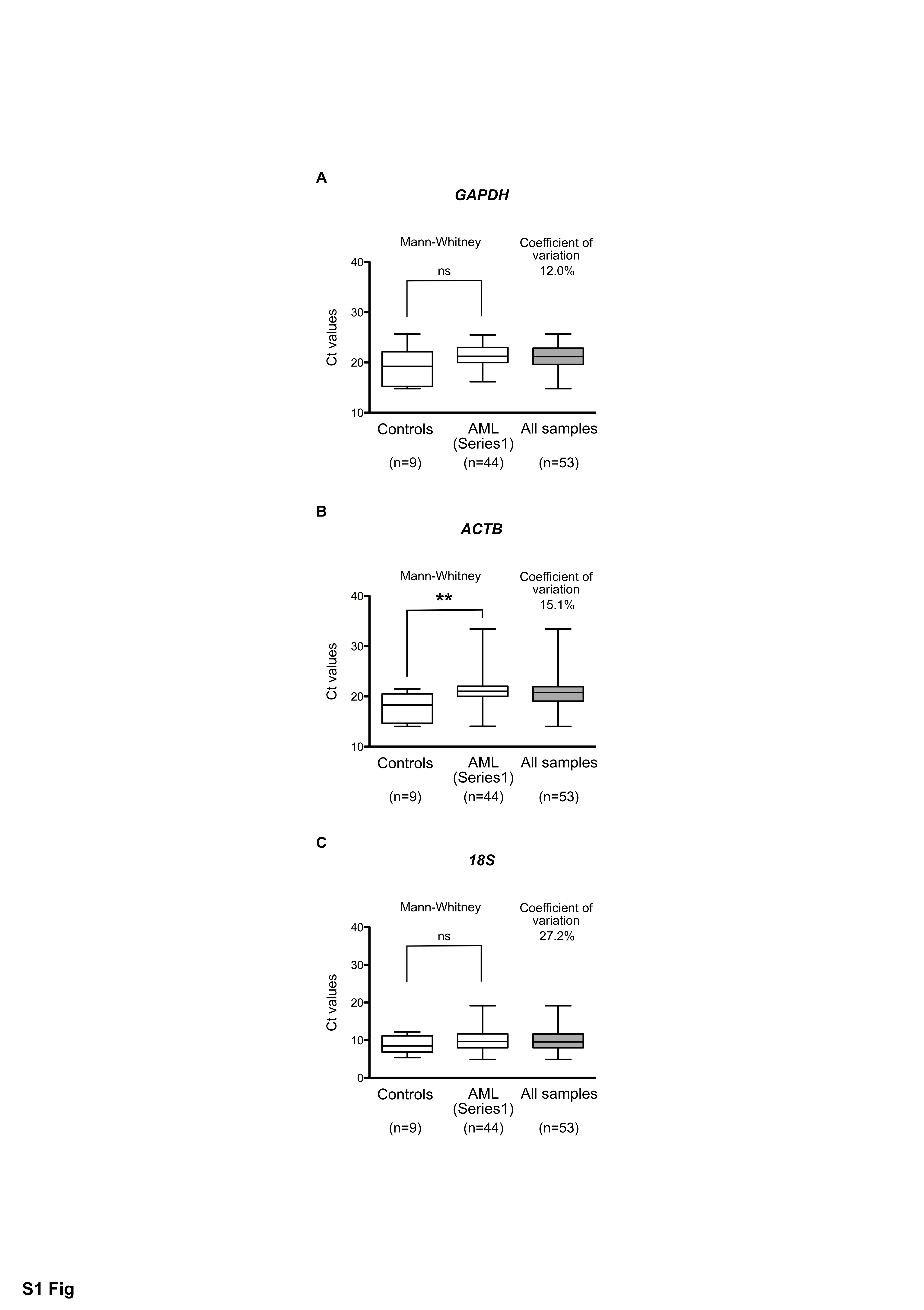

Supplement: S1 Fig — Ct values of three internal reference genes, GAPDH (A), ACTB (B) and 18S (C), were compared to identify the most suitable housekeeping gene in our experimental conditions. Three major criteria were required: first, the absence of significant difference between controls and AML patients; second, the smallest coefficient of variation; and third, a mean CT of about 20 that corresponds to the mean CT of most of our genes of interest. Only GAPDH gene matched these criteria and was thus used as normalizer. (TIFF) [file pone.0170160.s002.tiff]

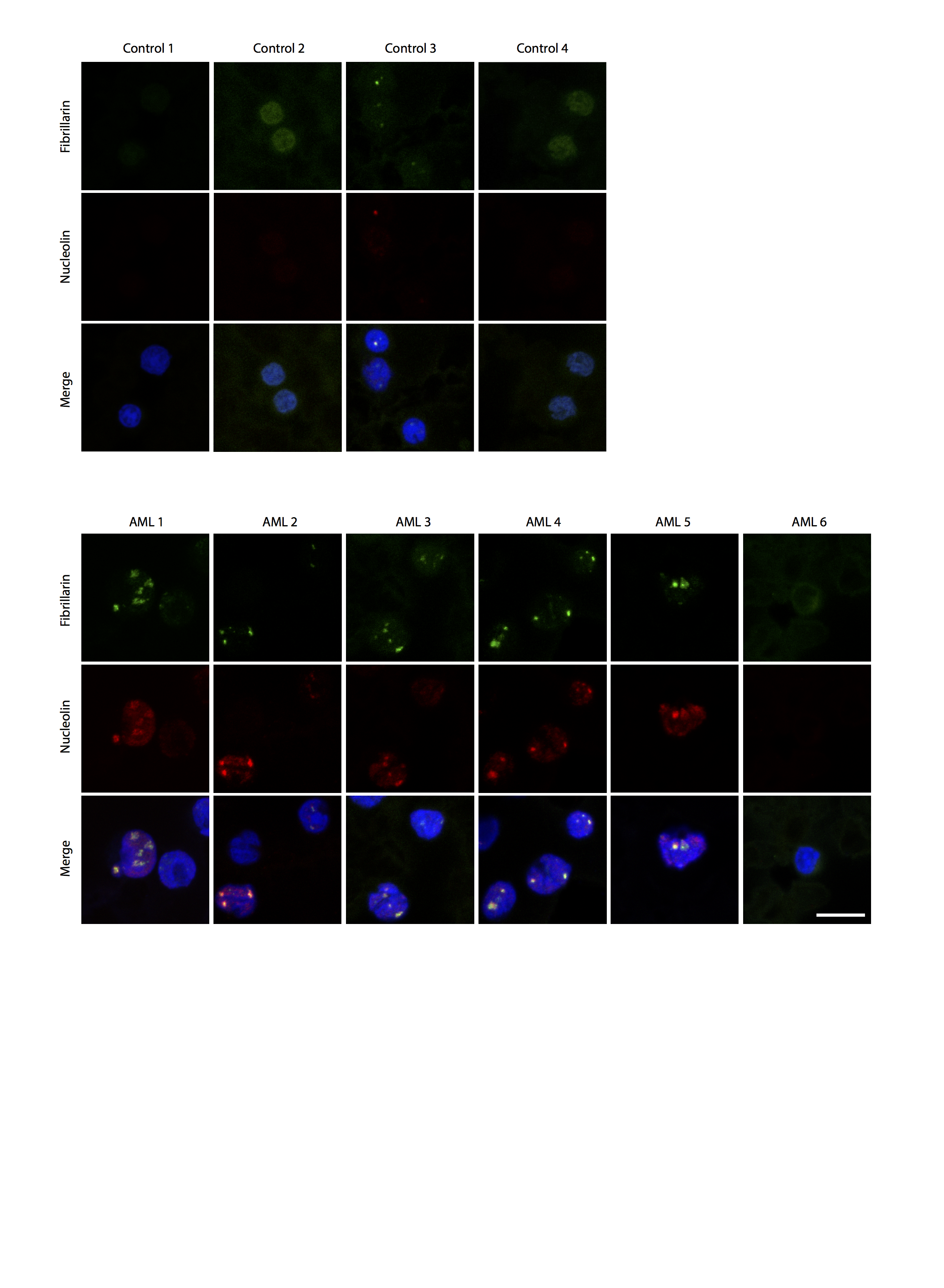

Supplement: S2 Fig — Immunofluorescence staining of nucleolar FBL and NCL on control (top panel) and AML (bottom panel) patient's bone marrow smears. FBL (green) and NCL (red) pattern are shown individually in top and middle images, and merged image with nuclei staining (blue) is shown in the bottom image. Images were collected using confocal microscopy (scale = 10μm). (TIFF) [file pone.0170160.s003.tiff]

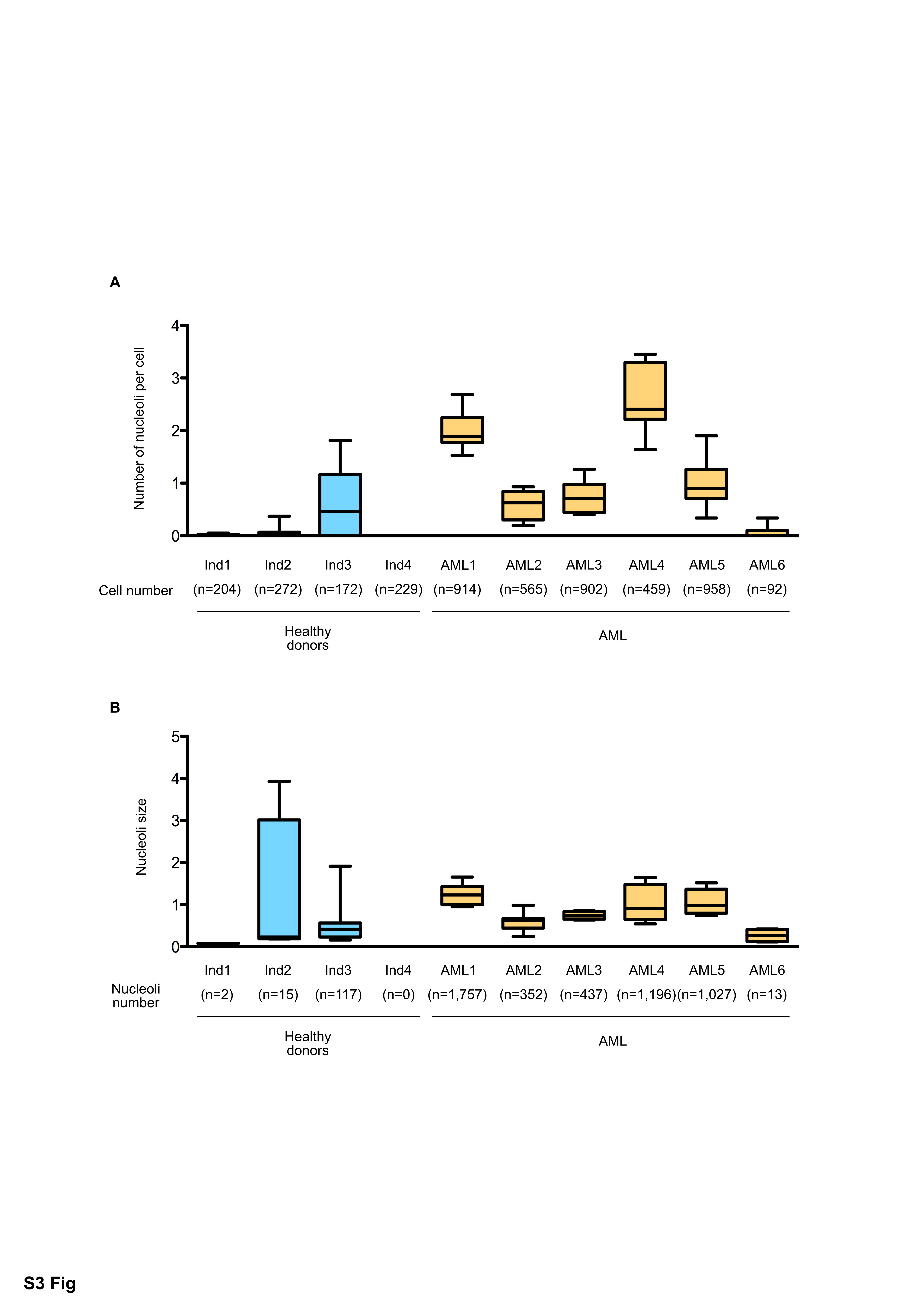

Supplement: S3 Fig — (A) Quantification and analysis of nucleoli number by image analysis from 4 control and 6 AML bone marrow smears. The number of nucleoli per cell was measured using FBL signal in each samples and ranged from 0 to 4. The median number of nucleoli per cell was represented for each individual samples. No nucleolus was observed in the control Ind4. (B) Quantification and analysis of nucleoli size by image analysis from 4 control and 6 AML bone marrow smears. When nucleoli are detected within a sample, the nucleolus size was measured using FBL signal in each samples. The median size of nucleoli per cell was represented for each individual samples. No nucleolus was observed in the control Ind4. Box and whisker plots represent median (middle bar in the box), interquartile range (bottom and top of box) and minimal/maximal values (bottom and top whisker). The number of cells or nucleoli analyzed for each samples are indicated in bracket. Representative panels of images used to perform these image analyses are shown in Fig 1 and S1 Fig. (TIFF) [file pone.0170160.s004.tiff]

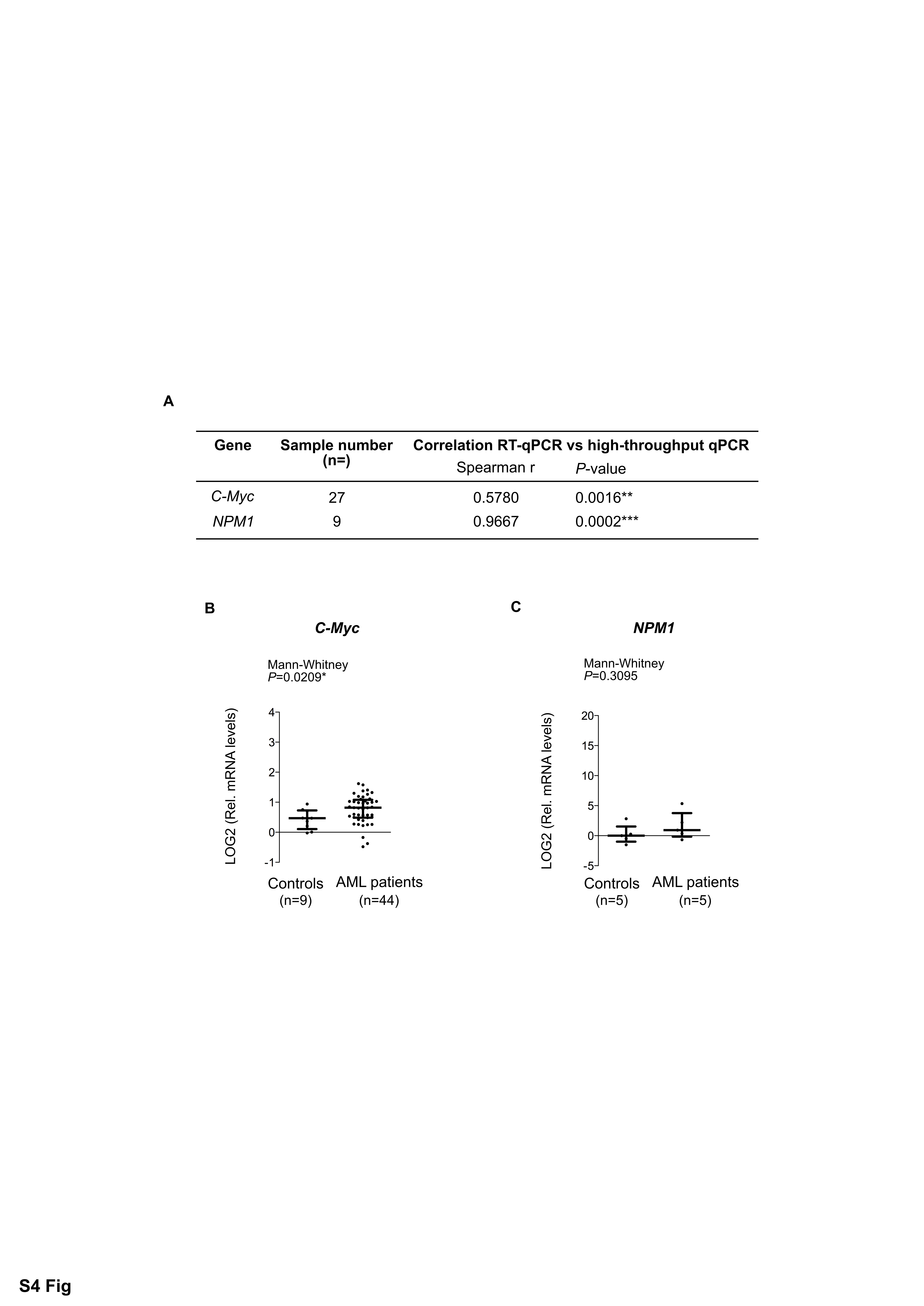

Supplement: S4 Fig — (A) Validation of high-throughput qPCR method using microfluidic system device. Correlation between fold-changes calculated from Ct values determined by classical RT-qPCR and high-throughput qPCR was investigated on few samples for C-Myc and NPM1 gene using Spearman test. (B-C) Expression of C-Myc and NPM1 genes in AML patients. Mean comparison of C-Myc (B) and NPM1 (C) gene expression between controls and AML patients was investigated using Mann-Whitney test in serie 1 analyzed by classical RT-qPCR. Graphs represents median (middle horizontal bar) and interquartile range (bottom and top bars) calculated on the Log2(Relative mRNA levels) of each individual samples (grey dot). n: number of samples; *: P<0.05; **: P<0.01; ***: P<0.001. (TIFF) [file pone.0170160.s005.tiff]

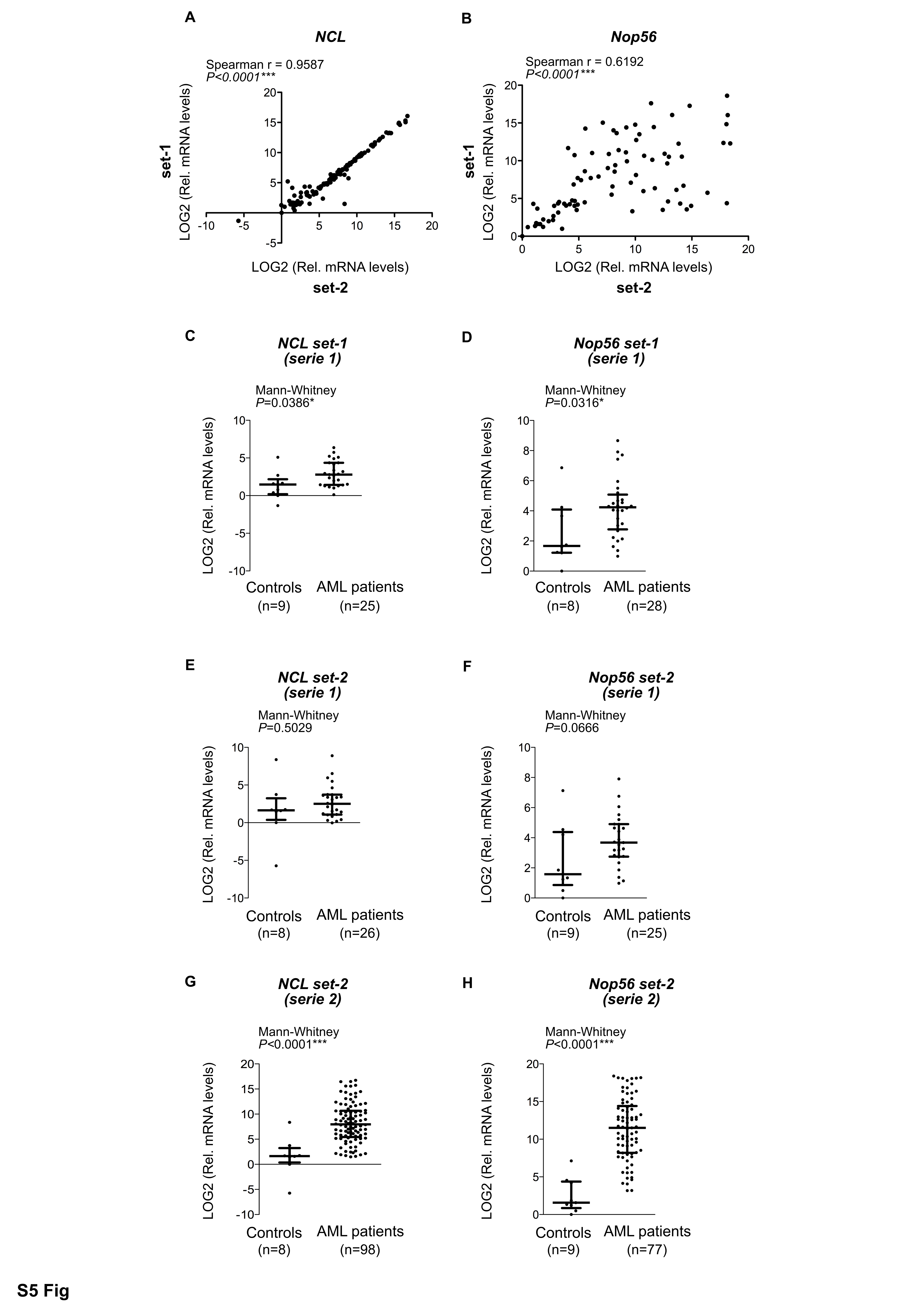

Supplement: S5 Fig — (A-B) Correlation between log2 (Relative mRNA levels) issued from two different sets of primers to analyze expression of NCL (A) and Nop56 (B). Correlation was determined using Spearman test. (C-H) Mean comparison of NCL and Nop56 gene expression between controls and AML patients using Mann-Whitey test. Relative RNA levels of NCL (C, E, G) and Nop56 (D, F, H) were determined using high-throughput qPCR in series 1 (C-F) and series 2 (G-H) with two different sets of primers (set-1: C, D; set-2: E-G, F-H). Graphs represents median (middle horizontal bar) and interquartile range (bottom and top bars) calculated on the log2 (Relative mRNA levels) of each individual samples (grey dot). n: number of samples; *: P<0.05; ***: P<0.0001. (TIFF) [file pone.0170160.s006.tiff]

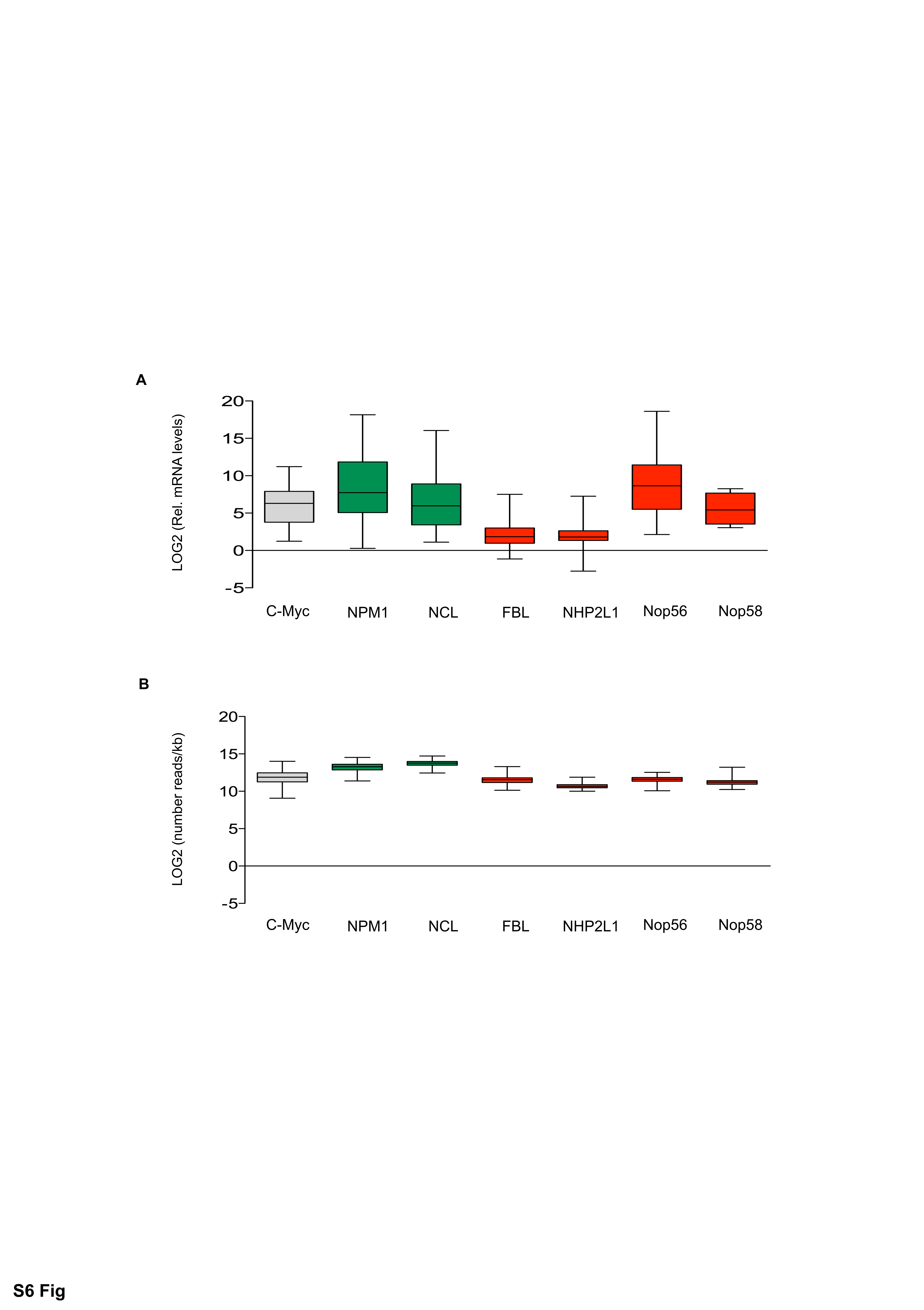

Supplement: S6 Fig — (A) Expression distribution of the different genes analyzed by high-throughput qPCR in series 3. (B) Expression distribution of the different genes analyzed by RNA-seq in series TCGA. Box and whisker plots represent median (middle bar in the box), interquartile range (bottom and top of box) and minimal/maximal values (bottom and top whisker). Grey: gene usually over-expressed in AML samples; green: genes coding proteins regulating rRNA synthesis; red: genes coding proteins of the rRNA methylation complex. (TIFF) [file pone.0170160.s007.tiff]

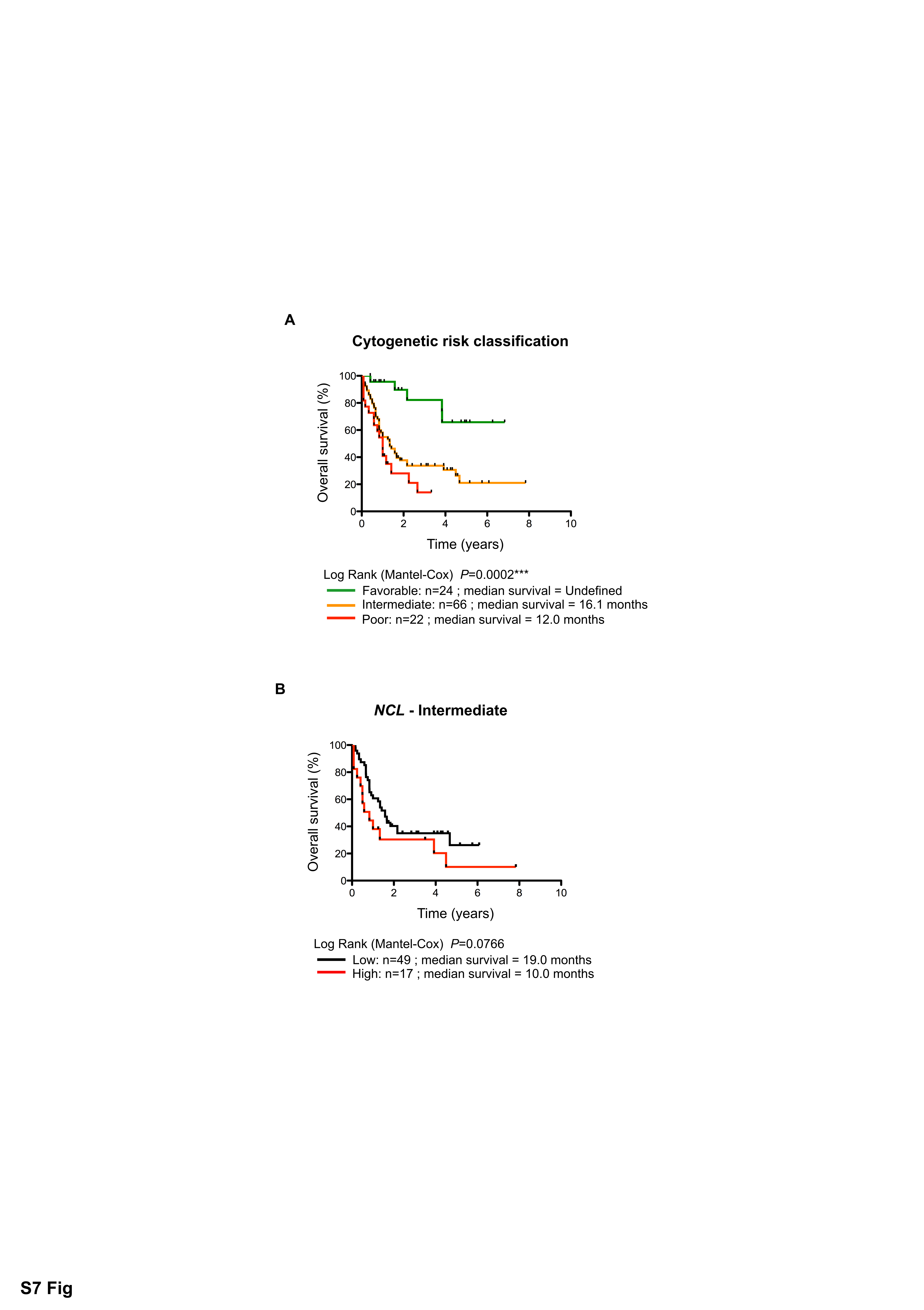

Supplement: S7 Fig — (A-B) Kaplan-Meier analysis of overall survival rates (event = death related to AML disease) according to cytogenetic risk classification (A) and to NCL in intermediate group of cytogenetic risk classification in AML patients (B). The data are dichotomized at the 75% percentile value into high and low mRNA level groups. n: number of samples. (TIFF) [file pone.0170160.s008.tiff]
