# Supplementary material for: Expression Profiling of Ribosome Biogenesis Factors Reveals Nucleolin as a Novel Potential Marker to Predict Outcome in AML Patients
Source: PLoS One. 2017 Jan 19;12(1):e0170160. doi: 10.1371/journal.pone.0170160 (PMC5245884; doi:10.1371/journal.pone.0170160)
Supplement: S1 File — (DOCX) [file pone.0170160.s009.docx]

**Supporting Information**

**Expression profiling of ribosome biogenesis factors reveals nucleolin as a novel potential marker to predict outcome in AML patients**

Virginie Marcel^1,2,3¶^, Frédéric Catez^1,2,3¶^, Caroline M. Berger^1,2,3^, Emeline Perrial^1,2,4^, Adriana Plesa^5^, Xavier Thomas^6^, Eve Mattei^5^, Sandrine Hayette^5^, Pierre Saintigny^1,2,7^, Philippe Bouvet^1,2,3,8^, Jean-Jacques Diaz^1,2,3&*^ and Charles Dumontet^1,2,4&*^

^1^ Cancer Research Center of Lyon, UMR INSERM 1052 CNRS 5286, Centre Léon Bérard, Lyon, France

^2^ Université Lyon 1, Lyon, France

^3^ Nuclear domains and pathologies team, Cancer Cell Plasticity Department, Lyon, France

^4^ Anticancer antibodies team, Immunity, Microenvironment and Virus Department, Lyon, France

^5^ Department of Biology, Hospices Civils de Lyon, Centre Hospitalier Lyon Sud, Pierre Bénite, France

^6^ Department of Hematology, Hospices Civils de Lyon, Centre Hospitalier Lyon Sud, Pierre Bénite, France

^7^ Department of Medecine, Centre Léon Bérard, Lyon, France

^8^ Ecole Normale Supérieure de Lyon, Lyon, France

^¶^ These authors contributed equally to this work as first authors.

^&^These authors contributed equally to this work as senior authors.

****Corresponding authors***:

E-mail: [charles.dumontet@chu-lyon.fr](mailto:charles.dumontet@chu-lyon.fr) (CD), jean-jacques.diaz@lyon.unicancer.fr (JJD)

**Supplementary Tables**

**Table A. Sequence of primer sets.**

| **Gene Name** | **Set** | **Direction** | **Sequence 5’ to 3’** |
| --- | --- | --- | --- |
| GAPDH | 1 | F | AGC-CAC-ATC-GCTCAG-ACA-C |
|  |  | R | GCC-CAA-TACGAC-CAA-ATC-C |
| C-Myc | 1 | F | CAC-CAG-CAGCGA-CTC-TGA |
|  |  | R | GAT-CCA-GACTCT-GAC-CTTTTG-C |
| NPM1 | 1 | F | TTG-TTG-AAG-CAGAGG-CAA-TG |
|  |  | R | TAT-TTC-AAAGCC-CCC-AAG-G |
| 18S | 1 | F | GTT-GGT-GGA-GCGATT-TGT-CT |
|  |  | R | GAA-CGC-CACTTG-TCC-CTC-TA |
| NCL | 1 | F | GTC-AGC-AAGGAT-GGG-AAA-AG |
|  |  | R | TAG-ATC-GCCCAT-CGA-TCT-CT |
|  | 2 | F | TCA-AGGT-GAC-CCC-AAG-AAA-A |
|  |  | R | GAC-GACC-TCT-TCT-CCA-CTG-C |
| FBL | 1 | F | CCT-GCG-TAA-TGGAGG-ACA-CT |
|  |  | R | GCT-GAG-GCTGTG-GAG-TCA-AT |
| NHP2L1 | 1 | F | GCT-ACT-GGA-CCTCGT-TCA-GC |
|  |  | R | ACT-CAG-AGATGC-CCC-TGT-TG |
| Nop56 | 1 | F | AGG-CTA-TTC-TGGATG-CCT-CA |
|  |  | R | GTA-GGC-TCTGGC-GGT-ATT-CA |
|  | 2 | F | GAG-GTG-CCCACG-AGT-GTA-TT |
|  |  | R | TGC-TTC-CTTCAT-GAC-ATC-CA |
| Nop58 | 1 | F | TGA-TGG-AGGGCA-AAA-TCA-AT |
|  |  | R | CGG-TTC-ATGGGC-TTC-TTT-TA |

**Table B. Comparison of gene expression in bone marrow samples vs blood samples in AML patients.**

| **Gene** | **Bone marrow** | | |  | **Blood** | | | **Mann-Whitney** |
| --- | --- | --- | --- | --- | --- | --- | --- | --- |
|  | **n (%)** | **Mean FC** | **SE** |  | **n (%)** | **Mean FC** | **SE** | ***P*-value^#^** |
| *C-Myc*  *NPM1*  *18S*  *NCL*  *FBL*  *NHP2L1*  *Nop56* | 13 (72.2)  61 (83.6)  27 (79.4)  70 (80.5)  65 (79.3)  70 (79.5)  76 (80.0) | 47.96  23.60  11.27  4.12  9.19  6.38  23.30 | 22.70  7.91  2.72  1.38  2.99  1.23  8.22 |  | 5 (27.8)  12 (16.4)  7 (20.6)  17 (19.5)  17 (20.7)  18 (20.5)  19 (20.0) | 7.68  12.06  16.02  1.58  12.75  12.55  10.54 | 2.61  7.98  5.38  0.82  8.97  8.36  5.51 | 0.11  0.62  0.16  0.61  0.27  0.65  0.49 |

*^#^*Two-tailed *P*-value

**Table C. Overall survival of AML patients related to expression of ribosome factors in series 3.**

|  | **Low** | |  | **High** | |  | **Log Rank (Mantel-Cox)** |
| --- | --- | --- | --- | --- | --- | --- | --- |
|  | **n** | **Median survival (months)** |  | **n** | **Median survival (months)** |  | **P-value** |
| **C-Myc** | 11 | 40.8 |  | 3 | Undefined |  | 0.6120 |
| **18S** | 18 | 18.2 |  | 5 | 12.6 |  | 0.0628 |
| **NPM1** | **36** | **20.9** |  | **11** | **15.8** |  | **0.0296*** |
| **NCL** | **45** | **20.9** |  | **14** | **15.8** |  | **0.0467*** |
| **FBL** | 38 | 23.5 |  | 13 | 16.2 |  | 0.7123 |
| **NHP2L1** | 36 | 20.9 |  | 13 | 13.1 |  | 0.3517 |
| **Nop56** | **44** | **23.5** |  | **15** | **13.6** |  | **0.0163*** |
| **Nop58** | 3 | 40.8 |  | 1 | Undefined |  | 0.3621 |

**Table D. Overall survival of AML patients related to expression of ribosome factors in series TCGA.**

| **Gene** | **Age** | **Low levels** | |  | **High levels** | |  | **Log Rank (Mantel-Cox)** |
| --- | --- | --- | --- | --- | --- | --- | --- | --- |
|  |  | **n** | **Median survival (months)** |  | **n** | **Median survival (months)** |  | ***P*-value** |
| **C-Myc** | All  < 60 years  ≥ 60 years | 85  46  39 | 22.1  56.1  12.0 |  | 28  13  15 | 14.0  54.0  10.0 |  | 0.2015  0.6328  0.2099 |
| **NPM1** | All | 85 | 19.0 |  | 28 | 26.0 |  | 0.7661 |
|  | < 60 years  ≥ 60 years | 43  42 | 56.1  12.0 |  | 16  12 | Undefined  9.0 |  | 0.6219  0.5532 |
| **NCL** | All | 85 | 20.0 |  | 28 | 10.0 |  | 0.0997 |
|  | < 60 years  ≥ 60 years | 43  **42** | 56.1  **15.0** |  | 16  **12** | 54.0  **6.0** |  | 0.4865  **0.0164*** |
| **FBL** | All  < 60 years  ≥ 60 years | 85  45  40 | 17.0  54.0  12.0 |  | 28  14  14 | 26.0  Undefined  8.5 |  | 0.3669  0.7094  0.3832 |
| **NHP2L1** | All | 85 | 20.0 |  | 28 | 15.0 |  | 0.6952 |
|  | < 60 years  ≥ 60 years | 42  43 | 56.1  12.0 |  | 17  11 | 54.0  8.0 |  | 0.7102  0.2388 |
| **Nop56** | All | 85 | 20.0 |  | 28 | 14.0 |  | 0.4399 |
|  | < 60 years  ≥ 60 years | 45  40 | 56.1  10.0 |  | 14  14 | 54.0  12.0 |  | 0.5754  0.4764 |
| **Nop58** | All | 85 | 19.0 |  | 28 | 14.0 |  | 0.5931 |
|  | < 60 years  ≥ 60 years | 44  41 | Undefined  303 |  | 15  10.0 | 54.0  14.0 |  | 0.1497  0.5008 |
|  | |  |  |  |  |  |  |  |

**Table E. Overall survival of AML patients related to expression of ribosome factors in series TCGA depending on cytogenetic risk classification.**

| **Gene** | **Cytogenetic risk classification** | **Low levels** | |  | **High levels** | |  | **Log Rank (Mantel-Cox)** |
| --- | --- | --- | --- | --- | --- | --- | --- | --- |
|  |  | **n** | **Median survival (months)** |  | **n** | **Median survival (months)** |  | ***P*-value** |
| **C-Myc** | Favorable  Intermediate  Poor | 18  56  10 | Undefined  16.1  19.5 |  | 6  10  12 | Undefined 15.9  11.0 |  | 0.3894  0.7944  0.4153 |
| **NPM1** | Favorable | 16 | Undefined |  | 8 | Undefined |  | 0.6049 |
|  | Intermediate  Poor | 50  19 | 17.0  12.0 |  | 16  22 | 12.0  Undefined |  | 0.5241  0.2266 |
| **NCL** | Favorable | 20 | Undefined |  | 4 | Undefined |  | 0.2349 |
|  | Intermediate  Poor | **49**  16 | **19.0**  12.0 |  | **17**  6 | **10.0**  8.5 |  | **0.0766**  0.2887 |
| **FBL** | Favorable  Intermediate  Poor | 15  52  18 | Undefined 15.9  12.0 |  | 9  14  4 | Undefined 23.0  6.5 |  | 0.9104  0.4956  0.5361 |
| **NHP2L1** | Favorable | 19 | Undefined |  | 5 | 26.0 |  | 0.1311 |
|  | Intermediate  Poor | 49  17 | 18.0  12.0 |  | 17  5 | 34.5  12.0 |  | 0.2790  0.3642 |
| **Nop56** | Favorable | 19 | Undefined |  | 5 | Undefined |  | 0.9944 |
|  | Intermediate  Poor | 49  **17** | 16.1  **12.0** |  | 17  **5** | 15.9  **7.0** |  | 0.8183  **0.0619** |
| **Nop58** | Favorable | 19 | Undefined |  | 5 | Undefined |  | 0.7684 |
|  | Intermediate  Poor | 48  18 | 17.0  12.0 |  | 18  4 | 11.0  12.0 |  | 0.4813  0.7653 |
|  | |  |  |  |  |  |  |  |
